# Lifestyle Factors and Inflammation: Associations by Body Mass Index

**DOI:** 10.1371/journal.pone.0067833

**Published:** 2013-07-02

**Authors:** Elizabeth D. Kantor, Johanna W. Lampe, Mario Kratz, Emily White

**Affiliations:** 1 Public Health Sciences Division, Fred Hutchinson Cancer Research Center, Seattle, Washington, United States of America; 2 Department of Epidemiology, University of Washington, Seattle, Washington, United States of America; Scientific Directorate, Bambino Hospital, Italy

## Abstract

Chronic inflammation, which is associated with obesity, may play a role in the etiology of several diseases. Thus, reducing inflammation may offer a disease-prevention strategy, particularly among the obese. Several modifiable factors have been associated with inflammation, including: dietary fiber intake, saturated fat intake, physical activity, smoking, alcohol, and use of certain supplements and medications (glucosamine, chondroitin, fish oil, vitamin E, statins and aspirin). To study whether these associations differ by body mass index (BMI), we used data on 9,895 adults included in the 1999–2004 cycles of the National Health and Nutrition Examination Survey (NHANES). Survey-weighted linear regression was used to evaluate the associations between modifiable factors and serum high-sensitivity C-reactive protein (hsCRP) concentrations across the following groups: underweight/normal weight (BMI<25 kg/m^2^), overweight (25-<30 kg/m^2^) and obese (30+ kg/m^2^). While several factors were significantly associated with decreased hsCRP among the normal weight or overweight groups (increased fiber intake, lower saturated fat intake, physical activity, not smoking, and use of chondroitin, fish oil and statins), only increasing dietary fiber intake and moderate alcohol consumption were associated with reduced hsCRP among the obese. Effect modification by BMI was statistically significant for the saturated fat-hsCRP and smoking-hsCRP associations. These results suggest that posited anti-inflammatory drugs and behaviors may be less strongly associated with inflammation among the obese than among lower weight persons.

## Introduction

Low-grade chronic inflammation has been associated with risk of several chronic diseases, including cardiovascular disease and several cancers[1]–[7], although its exact role in the etiology of these diseases is uncertain [8], [9]. It is therefore important to understand how one may reduce inflammation, as it is possible that reducing inflammation may represent a feasible disease-prevention strategy. Several modifiable factors have been associated with reduced inflammation, including: increased dietary fiber intake [10], decreased saturated fat intake [11], increased physical activity [12], not smoking [13], moderate alcohol consumption [14], and use of certain supplements and drugs: glucosamine [15], [16], chondroitin [15], [16], fish oil [15], [17], vitamin E [18], statins [19], and aspirin [20].

The question of how to reduce inflammation may be especially pertinent to obese individuals. Adipose tissue secretes pro-inflammatory cytokines, leading to a state of chronic low-grade inflammation associated with obesity, such that obese persons often experience higher concentrations of inflammatory biomarkers than their normal-weight counterparts [21]–[23]. Furthermore, obese individuals experience increased risk of chronic diseases with which inflammation has been implicated [24].

It is therefore plausible that those who are obese might reap most benefit from use of anti-inflammatory strategies. Yet, few studies have investigated whether the associations between these modifiable factors and inflammation vary according to obesity status. This study attempts to answer this question using data from the National Nutritional and Health Examination Survey (NHANES), with obesity measured by body mass index (BMI) and inflammation measured by serum high-sensitivity C-reactive protein (hsCRP).

## Methods and Procedures

### Ethics Statement

NHANES data are publicly available and are considered exempt by the University of Washington Institutional Review Board. All participants provided written informed consent and the survey was approved by the National Center for Health Statistics Institutional Review Board.

### Data Source/study Population

Data were used from the 1999–2000, 2001–2002, and 2003–2004 cycles of NHANES, as these cycles ascertained information on exposures, covariates, and outcome of interest. NHANES is a nationally-representative survey of civilian, non-institutionalized persons living in the United States [25]. This survey employs a stratified multi-stage probability design in which persons aged 60+ are oversampled, as are individuals of low income and certain racial/ethnic groups. Information on health and health-behaviors was collected at home interviews, with laboratory measures and additional data collected on a subset of participants.

Of the 13,876 persons aged 25+ included in these NHANES cycles, 12,063 had hsCRP measured and were therefore eligible for study. Women with positive/unknown pregnancy test results were excluded (n = 524), as were persons missing BMI (n = 356). Persons were also excluded if missing data on exposures, including: dietary data (ie, dietary fiber intake, saturated fat intake; n = 963), physical activity (n = 11), smoking (n = 19), supplement use (n = 73), statin use (n = 20), and aspirin use (n = 28). We also excluded persons missing information on covariates included in the analyses (n = 232). These exclusions are not mutually exclusive, as persons may have been removed for more than one reason. In order to remove persons who might be acutely ill while accounting for the fact that the appropriate definition of outlying values may vary according to personal characteristics, we further excluded persons with hsCRP values falling above the 98^th^ percentile for their age-gender-BMI group (n = 198) [26]. After making the above-listed exclusions, 9895 persons remained for analyses. For statin-specific analyses, 342 persons missing information on cholesterol were further excluded, leaving a total sample size of 9553. For analyses of alcohol, 1718 persons missing information on usual alcohol use were excluded, leaving 8177 persons for these analyses.

### Primary Exposures

As noted above, the primary exposures in this study are modifiable factors which have been associated with reduced inflammation, including: dietary fiber intake, saturated fat intake, physical activity, smoking, alcohol consumption, as well as use of glucosamine, chondroitin, fish oil, vitamin E, statins, and aspirin.

Dietary fiber intake and saturated fat intake were ascertained from 1 or 2 day recalls, with the second day included where available. Each recall ascertained dietary intake in the 24-hour period prior to interview (midnight-midnight) and was collected at either the time of examination or by telephone interview. The second day of recall was only collected during the 2003–2004 cycle and was available for approximately 32% of our sample. Dietary data for a given recall was excluded by NHANES if it was deemed unreliable according to their pre-set criteria; we further excluded men reporting energy intake <800 or >5000 kcal/day and women reporting <600 or >4000 kcal/day, as dietary intake on a day of abnormally high or low caloric intake likely would not be indicative of intake in the time frame relevant to the CRP measurement. The dietary fiber and saturated fat variables were created so as to best incorporate dietary recommendations, while also recognizing the need for sufficiently-sized groups. The daily reference value (DRV) for fiber is 25 grams/day [27], and we have therefore incorporated this threshold as one of our cut-points. We have further separated persons consuming less than 25 grams of fiber per day into the following three groups: ≤10 grams/day, >10-≤20 grams/day, and >20-≤25 grams/day. The 2010 USDA Dietary Guidelines for Americans recommends that less than 10% of calories be consumed from saturated fat, while also highlighting that consuming less than 7% of calories from saturated fat may further reduce risk of cardiovascular disease [28]. We have incorporated both of these cut-points into our saturated fat variable, which is categorized as follows: ≤7% kcal, >7-≤10% kcal, >10-≤13% kcal, and >13% kcal.

Physical activity was ascertained by a questionnaire administered at the time of household interview. Participants reporting any moderate or vigorous leisure-time physical activity (LTPA) in the last month were asked to report on usual frequency, duration, and intensity of various activities, such as swimming, running, and dance. For all moderate and vigorous activities, NHANES provided a corresponding Metabolic Equivalent of Task (MET)-score. From this information, we calculated average MET-hours per activity per week, which were then summed across activities for average MET-hours of LTPA per week. This physical activity variable was categorized into the following three groups: no leisure time physical activity, low activity (>0-<10 MET hrs/week), and high activity (≥10 MET hrs/week). This threshold of 10 MET hrs/week (or 600 MET minutes/week) was selected, as it falls within the 2008 Physical Activity Guidelines for Americans recommendation, which suggests that adults get 500–1000 MET-minutes of activity per week [29], while also allowing for sufficient sample size in both groups.

Persons never smoking or smoking less than 100 cigarettes in their lifetime were classified as never smokers, while those reporting more than 100 cigarettes were classified as either current or former smokers, depending on whether they reported current smoking at the time of interview. Alcohol consumption was ascertained from a questionnaire in which participants were asked how often they drank alcoholic drinks per week in the last year and, on average, how many drinks were consumed per occasion. From this data, average number of drinks per week was calculated and this variable was categorized as follows: 0 drinks/week, >0-≤3 drinks/week, >3-≤8 drinks/week, and >8 drinks/week. These categories were created so as to separate non-drinkers from drinkers, while also separating moderate alcohol consumption from low/high consumption, given that moderate alcohol consumption has been associated with reduced hsCRP [14]. A threshold of 8 drinks per week was selected to separate moderate drinkers from high drinkers, as it was the highest threshold we could set while maintaining adequate sample size in all groups.

Regular use of glucosamine, chondroitin, fish oil, and vitamin E supplements was ascertained from a detailed questionnaire of supplement use. Study participants were first asked if they used supplements in the 30 days prior to interview; those who reported use were then asked to provide the supplement name and usual frequency of use. To ascertain use of glucosamine, chondroitin, and fish oil, information on reported supplement brand name was linked to a NHANES database listing ingredients contained within each supplement formulation. Because vitamin E is often included in multivitamins and other supplements at low doses, we instead opted to use reported supplement name as an indication of biologically relevant vitamin E use. Each participant also listed usual frequency of use of each supplement, which was applied to each supplement to distinguish regular use from irregular use. Each supplement was modeled as a binary variable, with persons reporting use on 20+ days per month classified as regular users, while those reporting no use or use on <20 days/month were classified as non-users.

Statin use was determined by abstraction of statin medications from a list of all prescription medications used regularly in the prior 30 days, and was categorized as a binary variable (no/yes, with yes indicating regular use). Aspirin use was determined from a questionnaire specifically designed to assess history of prescription and over-the-counter of pain-reliever use, with pain-relievers of interest listed on a note card to aid in recall. From this data, we abstracted information on current use of aspirin-containing medications, with regular current use defined by report of current use daily or nearly every day; current regular aspirin use modeled as a binary no/yes variable.

### Body Mass Index

Height and weight were measured by NHANES staff, from which BMI was calculated (m/kg^2^). Five categories were used to model the main effect of BMI: underweight (BMI <18.5), normal weight (BMI 18.5-<25), overweight (BMI 25-<30), obese (BMI 30-<35), and severely obese (BMI 35+). We further collapsed BMI into three categories for assessment of stratum-specific associations and interaction: underweight/normal weight (BMI<25), overweight (BMI 25-<30), and obese (BMI 30+).

### Outcome

Our measure of inflammation, hsCRP, was measured by latex-enhanced nephelometry [30]. This assay has a lower detectable limit of 0.2 mg/L, and NHANES assigned a value of 0.1 mg/L to values falling below this level (n = 184). The hsCRP values were right-skewed and were therefore log-transformed for analysis. Values have been exponentiated for presentation.

### Covariates

We selected factors for adjustment *a priori*. All multivariate models include the following demographic factors: age (25–29, 30–39, 40–49, 50–59, 60–69, 70+), sex, race/ethnicity (non-Hispanic white, Mexican American, other Hispanic, non-Hispanic, and mixed race/other), education (less than high school, high school graduate/GED or equivalent, some college or associates degree, and college graduate/above). We further adjusted for quartiles of total energy intake (assessed via dietary recall) and BMI (as described in the Statistical Analysis section below). Multivariate models for a given exposure were also adjusted for the other exposures under study as potential confounders, including: dietary fiber intake, saturated fat intake, physical activity, smoking history, and use of chondroitin, fish oil, vitamin E, statins, and aspirin. Despite inclusion as a primary exposure, alcohol was not included as a covariate in models of other exposures since this variable was missing for 17% of our study population and a sensitivity analysis revealed that adjustment for alcohol made little difference. Furthermore, glucosamine use was also not included in multivariate model of other exposures, as glucosamine and chondroitin are often taken in a single pill and adjustment for the more strongly associated, chondroitin, was considered sufficient.

History of diabetes as diagnosed by a health professional (yes, no, borderline) and history of heart disease as diagnosed by a health professional (including coronary heart disease, angina, or myocardial infarction) were also included in multivariate models.

For analyses of supplements and medications, adjustment was also made for primary indications of use. Report of arthritis as diagnosed by a health-professional or joint pain in the absence of injury is a primary indication for use of glucosamine, chondroitin, fish oil, and aspirin use, and was therefore included in corresponding analyses. Similarly, adjustment was made for memory loss/confusion in analyses of fish oil and history of high cholesterol was included in analyses of statins.

### Statistical Analysis

Multivariate survey-weighted linear regression was used to account for the complex sampling strategy used in the collection of NHANES data. We have presented results in terms of exponentiated beta- coefficients, which represent the ratio of the geometric mean hsCRP among those exposed to those unexposed. Results between each exposure and hsCRP are presented within BMI strata (underweight/normal weight [BMI <25], overweight [BMI 25-<30], and obese [BMI >30]). Within each of these BMI-specific strata, we have additionally presented the adjusted geometric mean hsCRP corresponding to each level of exposure and have tested for trend or global association where applicable. The associations between each exposure of interest and hsCRP are adjusted for covariates described previously, as well as the expanded 5-level grouped linear variable BMI variable so as to reduce concern of residual confounding by BMI within the 3 broader strata. Specifically, within the underweight/normal weight group, we further adjusted for BMI<18.5 vs BMI 18.5-<25; similarly, within the obese group, we further adjust for BMI 30-<35 vs BMI 35+.

When testing for interaction across BMI strata, we created a single interaction term, with BMI treated as a 3-level grouped-linear variable and exposures of interest are similarly treated as grouped-linear variables. Two exceptions were alcohol and smoking, which were instead treated as indicator variables given *a priori* expectation of non-linear associations. Previous studies have found a J-shape relationship between alcohol and CRP [14] and we did not wish to assume a linear association between current-former-never smoking and CRP [13]. To fully control for confounding in our interaction models (which contain all three BMI strata), we also adjusted for confounder-BMI interactions observed to be significant in our analyses (BMI-smoking, BMI-saturated fat intake, BMI-age group, BMI-gender). All p-values presented are 2-sided.

Statistical analyses were conducted using Stata version 12 software (StataCorp IC, College Station, TX).

## Results

Of the 9,895 persons included in analyses, 2,968 were classified as underweight/normal weight, 3,696 were overweight, and 3,231 were obese. The unadjusted geometric mean of hsCRP within the underweight/normal weight group was 1.06 mg/L, while the unadjusted geometric mean hsCRP was 1.81 mg/L among the overweight and 3.68 mg/L among the obese.

As shown in Table 1, increasing age (*P*-trend: <0.001), BMI (*P*-trend <0.001), and percent of energy from saturated fat (*P*-trend 0.02) were associated with increasing hsCRP, while increasing education (*P*-trend: 0.04), fiber intake (*P*-trend: <0.001), and physical activity (*P-*trend: 0.001) were inversely associated with hsCRP. Smoking was associated with increased hsCRP (global *P*: <0.001), and alcohol intake was associated with hsCRP (global *P*: 0.04). Furthermore, females had higher hsCRP than males, and use of glucosamine, chondroitin, fish oil, and statins were all significantly associated with lower hsCRP. Vitamin E supplement use and aspirin use were not associated with hsCRP.

**Table 1:** Distribution of Demographic and Lifestyle Factors and Their Association with C-reactive Protein (hsCRP).

Factor	Raw Number^a^	Weighted Percent	Unadjusted Geometric Mean hsCRP (mg/L)	Multivariate Adjusted Ratio^b^	
				Ratio	95% CI
**Demographic Factors**					
**Age (years)**					
25–29	819	9.27	1.31	1.00	Ref
30–39	1,739	22.18	1.57	1.09	0.94, 1.25
40–49	1,937	23.47	1.77	1.17	1.05, 1.31
50–59	1,480	18.67	2.11	1.41	1.24, 1.60
60–69	1,783	13.49	2.64	1.70	1.46, 1.97
70+	2,137	12.92	2.42	1.77	1.53, 2.05
					P for trend: <0.001
**Gender**					
Male	4,948	48.68	1.57	1.00	Ref
Female	4,947	51.32	2.29	1.43	1.34, 1.53
**Race/Ethnicity**					
Non-Hispanic White	5,229	74.78	1.86	1.00	Ref
Mexican American	2,197	6.52	2.03	1.10	1.02, 1.19
Other Hispanic	428	4.98	1.91	1.04	0.93, 1.16
Non-Hispanic Black	1,734	9.53	2.38	1.01	0.93, 1.08
Other	307	4.18	1.56	0.97	0.83, 1.13
					Global P: 0.18
**Education**					
Less than High School Graduate	3,125	19.44	2.36	1.00	Ref
High School Graduate/GED	2,342	25.44	2.10	0.97	0.90, 1.04
Some College or AA Degree	2,492	28.85	1.91	0.94	0.87, 1.02
College Graduate or Above	1,936	26.27	1.47	0.93	0.86, 1.00
					P for trend: 0.04
**Lifestyle Factors**					
**Body Mass Index (kg/m^2^)**					
Underweight (<18.5)	135	1.65	0.75	1.00	Ref
Normal Weight (18.5- <25)	2,833	31.06	1.08	1.50	1.15, 1.95
Overweight (25- <30)	3,696	35.71	1.81	2.53	1.92, 3.33
Obese (30- <35)	1,960	19.09	2.94	3.91	2.98, 5.14
Severely Obese (35+)	1,271	12.49	5.21	6.60	5.01, 8.71
					P for trend: <0.001
**Dietary Fiber Intake (grams/day)**					
≤10	2,773	27.05	2.31	1.00	Ref
>10- ≤20	4,604	46.93	1.94	0.90	0.83, 0.96
>20- ≤25	1,132	12.11	1.71	0.85	0.76, 0.96
>25	1,386	13.92	1.35	0.73	0.66, 0.81
					P for trend: <0.001
**Saturated Fat Intake (% total energy)**					
≤7	1,464	13.82	1.63	1.00	Ref
>7- ≤10	2,926	27.37	1.73	1.00	0.92, 1.09
>10 - ≤13	3,137	32.37	1.97	1.08	1.00, 1.18
>13	2,368	26.45	2.19	1.09	0.99, 1.21
					P for trend: 0.02
**Leisure Time Physical Activity (MET-hours/week)**					
None	4,434	36.47	2.41	1.00	Ref
Low (>0- <10)	2,152	24.42	1.91	0.91	0.85, 0.98
High (≥10)	3,309	39.11	1.53	0.88	0.82, 0.94
					P for trend: 0.001
**Smoking**					
Current	2,106	23.07	2.02	1.00	Ref
Former	2,875	27.73	1.98	0.84	0.78, 0.90
Never	4,914	49.20	1.81	0.82	0.76, 0.88
					Global P: <0.001
**Alcohol Use (drinks/week)**					
0	2,064	21.02	2.47	1.00	Ref
>0- ≤3	4,054	50.61	1.83	0.96	0.89,1.04
>3- ≤8	1,088	14.74	1.41	0.87	0.77, 0.97
>8	971	13.63	1.70	1.01	0.90, 1.14
					Global P: 0.04
**Supplement/Drug Use**					
**Glucosamine (regular use)** c,d					
No	9,534	95.84	1.90	1.00	Ref
Yes	361	4.16	1.89	0.87	0.78, 0.98
**Chondroitin (regular use)** c,d					
No	9,643	97.19	1.91	1.00	Ref
Yes	252	2.81	1.75	0.82	0.71, 0.95
**Fish Oil (regular use)** c,e					
No	9,728	97.84	1.91	1.00	Ref
Yes	167	2.16	1.49	0.86	0.74, 1.00
**Vitamin E (regular use)** c					
No	8,807	88.10	1.91	1.00	Ref
Yes	1,088	11.90	1.84	0.95	0.88, 1.03
**Statins** f					
No	8,382	88.75	1.87	1.00	Ref
Yes	1,171	11.25	2.16	0.86	0.78, 0.94
**Aspirin** d,g					
No	8,589	87.56	1.85	1.00	Ref
Yes	1,306	12.44	2.33	0.99	0.92, 1.07

Abbreviations: hsCRP, high-sensitivity C-reactive protein.

a All variables total to 9895, except for alcohol use (n = 8177 ) and statin use (n = 9553).

b Adjusted for age, gender, race/ethnicity, education, smoking history (current, former, never), body mass index, physical activity, vitamin E supplement use, dietary fiber intake, dietary saturated fat intake, total energy intake, aspirin use, non-aspirin NSAID use, statin use, diabetes, coronary artery disease, regular use of glucosamine, chondroitin, fish oil, and vitamin E supplements, as well as age group*BMI group, gender*BMI group, saturated fat*BMI group, and smoking history group*BMI group interactions, with BMI group defined as three level grouped linear variable (underweight/normal weight, overweight, and obese).

c Regular use defined as use in the past 30 days with reported frequency of use of 20+ days/month.

d Multivariate model additionally adjusted for arthritis and/or joint pain not caused by injury.

e Multivariate model additionally adjusted for arthritis and/or joint pain not caused by injury and memory loss/confusion.

f Statin use defined as use of a statin drug among persons who report use of medication in the past month for which prescription was needed; analyses of statins further adjusted for cholesterol level.

g Aspirin use defined by use of the product every day or nearly every day in the last 30 days among those who report use of pain relievers taken nearly every day for a month or longer.

Increasing dietary fiber intake was associated with significantly lower hsCRP among the underweight/normal weight (*P*-trend: 0.05), overweight (*P*-trend <0.001), and obese groups (*P*-trend: 0.009) (Table 2). Increasing saturated fat intake was associated with increased hsCRP among the underweight/normal weight (*P*-trend: 0.04), but not among the overweight or obese. Increasing physical activity was associated with reduced hsCRP among the underweight/normal weight (*P*-trend: 0.05) and overweight (*P*-trend: 0.01), but not among the obese.

**Table 2:** Association of Inflammation Related Factors with C-reactive Protein (hsCRP), Stratified by Body Mass Index.

	Under−/Normal Weight (BMI <25); n = 2968				Overweight (BMI 25-<30); n = 3696				Obese (BMI 30+); n = 3231				
	Unadjusted Geometric Mean hsCRP: 1.06 mg/L				Unadjusted Geometric Mean hsCRP: 1.81 mg/L				Unadjusted Geometric Mean hsCRP: 3.68 mg/L				
Factor	Raw Number^a^	Adjusted Geometric Mean hsCRP (mg/L)	Multivariate Adjusted^b^		Raw Number^a^	Adjusted Geometric Mean hsCRP (mg/L)	Multivariate Adjusted^b^		Raw Number^a^	Adjusted Geometric Mean hsCRP (mg/L)	Multivariate Adjusted^b^		*P* c
			Ratio	95% CI			Ratio	95% CI			Ratio	95% CI	
***Lifestyle Factors***													
**Dietary Fiber (grams/day)**													
≤10	828	1.18	1.00	Ref	951	2.09	1.00	Ref	994	4.02	1.00	Ref	0.99
>10- ≤20	1,364	1.05	0.89	0.77, 1.02	1,727	1.86	0.89	0.80, 0.99	1,513	3.69	0.92	0.81, 1.04	
>20- ≤25	324	1.06	0.90	0.69, 1.16	446	1.83	0.88	0.74, 1.04	362	3.17	0.79	0.66, 0.95	
>25	452	0.93	0.79	0.64, 0.97	572	1.31	0.63	0.55, 0.71	362	3.38	0.84	0.72, 0.99	
				*P for trend: 0.05*				*P for trend: <0.001*				*P for trend: 0.009*	
**Dietary Saturated Fat (% kcal)**													
≤7	504	0.96	1.00	Ref	561	1.77	1.00	Ref	399	3.65	1.00	Ref	0.05
>7- ≤10	884	1.01	1.05	0.88, 1.24	1,147	1.69	0.95	0.84, 1.08	895	3.63	1.00	0.84, 1.19	
>10 - ≤13	907	1.10	1.14	0.95, 1.38	1,175	1.93	1.09	0.97, 1.23	1,055	3.63	0.99	0.84, 1.18	
>13	673	1.17	1.22	1.00, 1.47	813	1.83	1.03	0.91, 1.18	882	3.80	1.04	0.86, 1.26	
				*P for trend: 0.04*				*P for trend: 0.18*				*P for trend: 0.54*	
**Leisure Time Physical Activity**													
None	1250	1.16	1.00	*Ref*	1594	2.06	1.00	Ref	1590	3.72	1.00	Ref	0.46
Low (>0- <10 MET-hr/week)	630	1.03	0.89	0.77, 1.03	800	1.65	0.80	0.71, 0.90	722	3.94	1.06	0.95, 1.18	
High (≥10 MET-hr/week)	1088	1.01	0.88	0.77, 0.99	1302	1.72	0.83	0.73, 0.95	919	3.43	0.92	0.83, 1.02	
				*P for trend: 0.05*				*P for trend: 0.01*				*P for trend: 0.13*	
**Smoking**													
Current	804	1.27	1.00	Ref	713	2.16	1.00	Ref	589	3.84	1.00	Ref	0.03
Former	719	1.08	0.84	0.71, 0.99	1177	1.70	0.78	0.68, 0.89	979	3.64	0.96	0.85, 1.08	
Never	1445	0.95	0.75	0.63, 0.88	1806	1.74	0.79	0.69, 0.89	1663	3.65	0.96	0.87, 1.07	
				*Global P: 0.003*				*Global P: 0.001*				*Global P: 0.73*	
**Alcohol Use (drinks/week)**													
0	519	1.06	1.00	Ref	738	1.83	1.00	Ref	807	3.83	1.00	Ref	0.28
>0- ≤3	1248	1.04	1.01	0.83, 1.24	1489	1.85	0.99	0.89, 1.11	1317	3.48	0.91	0.84, 0.99	
>3- ≤8	380	1.03	1.02	0.80, 1.30	431	1.51	0.82	0.70, 0.97	277	3.04	0.80	0.68, 0.94	
>8	323	1.22	1.19	0.88, 1.61	419	1.80	0.98	0.82, 1.17	229	3.52	0.92	0.78, 1.10	
				*Global P: 0.66*				*Global P: 0.07*				*Global P: 0.05*	
***Supplement/Drug Use***													
**Glucosamine (regular use)** d,e													
No	2875	1.07	1.00	Ref	3560	1.82	1.00	Ref	3099	3.69	1.00	Ref	0.45
Yes	93	0.85	0.79	0.60, 1.04	136	1.59	0.88	0.71, 1.09	132	3.53	0.96	0.81, 1.13	
**Chondroitin (regular use)** d,e													
No	2898	1.07	1.00	Ref	3599	1.82	1.00	Ref	3146	3.70	1.00	Ref	0.89
Yes	70	0.83	0.77	0.61, 0.98	97	1.56	0.86	0.67, 1.10	85	3.13	0.85	0.67, 1.06	
**Fish Oil (regular use)** d,f													
No	2917	1.07	1.00	Ref	3264	1.81	1.00	Ref	3187	3.69	1.00	Ref	0.26
Yes	51	0.80	0.75	0.59, 0.94	72	1.67	0.92	0.70, 1.22	44	3.30	0.89	0.72, 1.12	
**Vitamin E (regular use)** d													
No	2587	1.07	1.00	Ref	3278	1.81	1.00	Ref	2942	3.72	1.00	Ref	0.78
Yes	381	1.00	0.93	0.79, 1.09	418	1.82	1.01	0.90, 1.12	289	3.39	0.91	0.80, 1.04	
**Statins** g													
No	2602	1.06	1.00	Ref	3060	1.86	1.00	Ref	2720	3.74	1.00	Ref	0.31
Yes	250	1.02	0.97	0.74, 1.26	506	1.48	0.79	0.68, 0.92	415	3.31	0.88	0.76, 1.03	
**Aspirin** e,h													
No	2649	1.07	1.00	Ref	3163	1.80	1.00	Ref	2777	3.70	1.00	Ref	0.67
Yes	319	1.03	0.97	0.78, 1.20	533	1.85	1.03	0.89, 1.18	454	3.58	0.97	0.87, 1.08	

Abbreviations: BMI, body mass index; hsCRP, high-sensitivity C-reactive protein.

a Persons missing information on cholesterol levels were excluded from analyses of statins on hsCRP: underweight/normal weight (n = 2852), overweight (n = 3566), obese (n = 3135); persons missing information on alcohol levels were excluded from analyses of alcohol on hsCRP, leaving sample sizes as follows: underweight/normal weight (n = 2470), overweight (n = 3077), obese (n = 2630).

b Adjusted for age, gender, race/ethnicity, education, smoking history, body mass index, physical activity, vitamin E supplement use, dietary fiber intake, dietary saturated fat intake, total energy intake, aspirin use, non-aspirin NSAID use, statin use, diabetes, coronary artery disease, regular use of glucosamine, chondroitin, fish oil, and vitamin E supplements, as well as continuous age group-BMI group, gender-BMI group, saturated fat*BMI group, and smoking history-BMI group interactions, with BMI group defined as three level grouped linear variable (underweight/normal weight, overweight, and obese).

c P for interaction.

d Regular use defined as use in the past 30 days with reported frequency of use of 20+ days/month.

e Multivariate model additionally adjusted for arthritis and/or joint pain not caused by injury.

f Multivariate model additionally adjusted for arthritis and/or joint pain not caused by injury and memory loss/confusion.

g Statin use defined as use of a statin drug among persons who report use of medication in the past month for which prescription was needed; analyses of statins further adjusted for cholesterol levels.

h Aspirin use defined by use of the product every day or nearly every day in the last 30 days among those who report use of pain relievers taken nearly every day for a month or longer.

Among underweight/normal weight persons, both former and never smokers had lower hsCRP than current smokers (global *P*: 0.003): former smokers had 16% lower hsCRP (Ratio: 0.84; 95% CI: 0.71,0.99) than current smokers, while never smokers had 25% lower hsCRP (Ratio: 0.75; 95% CI: 0.63,0.88). Similarly, among overweight individuals, former smokers and never smokers had lower hsCRP than current smokers (global *P*: 0.001), while no association was observed between smoking and hsCRP among the obese. There was no evidence of global association between number of drinks consumed per week and hsCRP among the normal weight group, though a significant association was observed between alcohol intake and hsCRP among obese individuals (global *P*: 0.05), with persons consuming >3–8 drinks per week experiencing a statistically significant 20% lower hsCRP than persons reporting no alcohol intake (Ratio: 0.80; 95% CI: 0.68,0.94). Among the overweight group, the association between alcohol intake and hsCRP approached statistical significance (p = 0.07).

Among the underweight/normal weight, regular users of chondroitin supplements had 23% lower hsCRP than non-users (Ratio: 0.77; 95% CI: 0.61,0.98) and regular use of fish oil was associated with 25% lower hsCRP (Ratio: 0.75; 95% CI: 0.59,0.94). However, for both chondroitin and fish oil, no significant association was observed among the overweight and obese. As compared to non-use, statin use was associated with 21% lower hsCRP among the overweight (Ratio: 0.79; 95% CI: 0.68,0.92), with no significant association observed among the normal weight and obese.

Among the differences between BMI groups in factors associated with hsCRP noted above, only two reached statistical significance for the test for interaction by BMI: the saturated fat–hsCRP association (*P-*interaction: 0.05) and the smoking-hsCRP association (*P*-interaction: 0.03).

We did not observe aspirin use or vitamin E supplement use to be associated with hsCRP in the overall analysis (Table 1), nor did we observe these factors to be significantly associated with hsCRP within BMI-specific strata.

## Discussion

In this study, several factors were significantly associated with hsCRP among normal weight and overweight persons, while only increasing dietary fiber intake and moderate alcohol consumption were significantly associated with reduced hsCRP among obese persons. The BMI-smoking and BMI-saturated fat intake interactions were both statistically significant, though interactions between BMI and other modifiable factors did not reach statistical significance.

Increasing fiber intake was significantly associated with reduced hsCRP in all three BMI groups, with no evidence of interaction. Little previous research has assessed whether BMI may act to modify the association between fiber intake and inflammation, though in a cross-sectional study of 1060 adults aged 18 and older, Oliveira *et al*. also observed that BMI did not significantly modify the association between fiber intake and CRP [31]. Animal models suggest several mechanisms by which higher fiber intake may reduce systemic inflammation. Gut microbes convert fiber to short chain fatty acids, which have been suggested to reduce intestinal permeability and uptake of gut microbiota-derived bacterial antigens including lipopolysaccharide, an endotoxin known to instigate inflammatory response [32], [33]. Furthermore, one of these short chain fatty acids, butyrate, is thought to reduce inflammation via direct inhibition of transcription factor nuclear factor kappa B (NFkB) [34], [35]. Percent of energy intake from saturated fat was positively associated with hsCRP among normal weight persons in our study, but not among overweight or obese groups. Certain saturated fatty acids have been shown to contribute to inflammation via increased activity of NFkB [36]. Increased fat intake may also alter the composition of the gut microbiota or increase intestinal permeability, both of which may increase the uptake of lipopolysaccharide or other bacterial antigens [37], [38]. It is not clear why the association between saturated fat intake and hsCRP may be stronger among persons of lower BMI, though it is notable that the composition of the gut microbiota is thought to vary with obesity [35].

Increasing physical activity was associated with reduced hsCRP in all three BMI groups, though the association was only statistically significant in the normal weight and overweight groups. However, randomized trials have demonstrated that exercise intervention significantly reduces CRP among the obese, with the reduction in CRP independent of weight loss [39]–[41]. Mechanisms by which physical activity may reduce systemic inflammation include increased insulin sensitivity and reduced diurnal insulin concentrations, which in and of itself may affect inflammatory processes [12].

We found evidence of a significant interaction between smoking history and BMI, with underweight/normal weight and overweight former and never smokers experiencing lower hsCRP than current smokers. It is thought that smoking may affect systemic inflammation through an oxidative stress pathway [13]. It is not clear why smoking may be more strongly associated with hsCRP among persons of lower BMI.

We found evidence of an association between alcohol intake and hsCRP among the obese, but not among normal weight individuals. Oliveira and colleagues also observed a stronger association between alcohol intake and CRP among men of higher BMI than among men of lower BMI, though the opposite pattern was observed among women [42]. It is hypothesized that moderate alcohol consumption inhibits adipocyte secretion of interleukin-6 (IL-6) [43].

Among normal weight individuals, use of chondroitin and fish oil supplements was associated with lower hsCRP concentrations, with weaker non-significant associations observed among overweight or obese individuals. We know of no other studies that assessed the interaction between use of chondroitin and BMI on hsCRP. Laboratory evidence suggests that chondroitin may reduce inflammation via inhibition of NFkB [44]. While the interaction between omega-3 polyunsaturated fatty acid (PUFA)-containing fish oil supplements and BMI on hsCRP has not been reported on previously, the interaction between blood measures of omega-3 PUFAs, eicosapentanoic acid (EPA) and docosahexanoic acid (DHA), and BMI on CRP has been studied. In a cross-sectional study of 330 Alaskan natives, Makhoul and colleagues observed that the inverse association between CRP and red blood cell EPA and DHA was stronger among heavier groups [45]. This finding closely mimics that of Klein-Platat *et al*., in which a stronger association was observed between blood levels of EPA and CRP among overweight adolescents than among normal weight adolescents [46]. While these two findings differ from those of the current study, it is possible that such differences may reflect different choice in omega-3 measures or differences in populations studied. Omega-3 PUFAs contained within fish oil supplements are thought to reduce inflammation in several ways, including inhibition of NFkB activation, competition with pro-inflammatory omega-6 PUFAs for the cyclooxygenase enzyme, and displacement of omega-6 from cell membrane stores [47].

In our study, we observed statin use to be associated with lower hsCRP among overweight and obese individuals, though the association among the obese was weaker and not statistically significant. In a randomized control trial (RCT), Nicholls *et al*. reported intensive atorvastatin treatment to be associated with a greater percent change in CRP among persons with BMI at or above median than among persons below the median, though this pattern was not observed with moderate pravastatin treatment [48]. In a trial of 48 obese individuals, Chan *et al.* reported that 6 weeks of atorvastatin treatment significantly reduced CRP and IL-6 [49]. It remains unclear why we observed a weaker association among the obese than among the overweight in our study and why results among the obese were weaker than those of the Chan trial. Research suggests that statins may reduce inflammation through inhibition of NFkB [50].

It is not clear why more factors were observed to be significantly associated with reduced hsCRP among normal or overweight individuals than among obese individuals. In addition to potential biologic differences between obese and normal weight individuals, it may be that the much higher level of inflammation among obese individuals makes reduction of inflammation relatively intractable. Adipose tissue is a major contributing factor to systemic inflammation, with adipose tissue generating approximately one third of the circulating pro-inflammatory cytokine IL-6 [21], with estimates likely varying across obesity status. IL-6 stimulates production of CRP in the liver [51], so it is plausible that the adipose tissue of the obese is contributing such a high level of hsCRP that other lifestyle factors do not make a substantial relative contribution. It is also possible that we have more measurement error among the obese for certain exposures, as obese persons report physical activity and dietary intake, particularly intake of fatty foods, with more error than non-obese persons [52]–[55]. Such measurement error may attenuate associations among obese individuals, potentially obscuring some of the associations among that group.

Advantages of this study include its large sample size, which has enabled the study of interaction. This study also has several limitations. First, as with any observational study, we cannot preclude the presence of unmeasured or residual confounding. Second, there is likely measurement error of exposures, which would attenuate results within BMI strata. For example, we were unable to discern high-dose versus low-dose aspirin intake. Furthermore, self-reported physical activity has generally not correlated well with doubly-labeled water, an objective measure of energy expenditure [56] and estimates of self-reported physical activity do not align well with those from accelerometry [57]. Third, we may have had limited power to detect significant interaction for the less common exposures. Finally, we decided *a priori* to analyze data using log-transformed hsCRP, with results reported on the relative scale. This scale accommodates variables with substantial numbers of observations with values close to zero, while an additive scale does not. For example, among the overweight group, which had a geometric mean of hsCRP of 1.81 mg/L, we observed a 0.8 mg/L difference between those in the highest vs. lowest fiber intake groups; while for the normal weight group, with mean hsCRP of 1.06 mg/L, this magnitude difference would be highly unlikely to be achieved. However, we also present adjusted geometric means for each level of each lifestyle factor within each BMI group, so that absolute differences in hsCRP can be seen.

In conclusion, this study suggests that the posited anti-inflammatory factors are less associated with hsCRP levels among obese individuals than among normal weight or overweight individuals. Only two of the factors studied were significantly associated with hsCRP among the obese, while several factors were associated with reduced inflammation among the normal weight and overweight groups. Despite a similar pattern observed across several factors, our conclusions are tempered by the fact that only two interactions were observed to be statistically significant, and for some exposures, there may be more measurement error among the obese. Considering effects of anti-inflammatory modalities by obesity status may become increasingly important as we attempt to identify ways of reducing inflammation and the burden of diseases with which inflammation has been implicated, especially among obese individuals.
